# Predictive value of TCCD and regional cerebral oxygen saturation for detecting early postoperative brain injury

**DOI:** 10.1007/s10877-024-01165-y

**Published:** 2024-05-17

**Authors:** Yu Liu, Lin Zhao, Xinlei Wang, Zhouquan Wu

**Affiliations:** 1 Graduate School of Dalian Medical University, Liaoning, 116044 China; 2 Department of Anesthesiology, Nanjing Medical University Affiliated, Changzhou No. 2 People’s Hospital, Changzhou, 213003 China

**Keywords:** TCCD, Regional cerebral oxygen saturation, Cardiovascular surgery, Brain injury

## Abstract

**Objective:**

This study aims to analyze the risk factors for early postoperative brain injury in patients undergoing cardiovascular surgery and explore the predictive value of transcranial color Doppler (TCCD) and regional cerebral oxygen saturation (rSO_2_) for detecting early postoperative brain injury in cardiovascular surgery patients.

**Methods:**

A total of 55 patients undergoing cardiovascular surgery with cardiopulmonary bypass in Changzhou No.2 The People’s Hospital of Nanjing Medical University were included in this study. Neuron-specific enolase (NSE) concentration was measured 24 h after operation. Patients were divided into brain injury (NSE ≥ 16.3 ng/mL) and normal (0 < NSE < 16.3 ng/mL) groups according to the measured NSE concentration. The clinical outcomes between the two groups were compared, including decreased rSO_2_ and cerebral blood flow (as measured by TCCD) levels. The risk factors of early postoperative brain injury were analyzed by multivariate logistic regression analysis, and the significant variables were analyzed by receiver operating characteristic (ROC) analysis.

**Results:**

A total of 50 patients were included in this study, with 20 patients in the brain injury group and 30 patients in the normal group. Cardiopulmonary bypass time (min) (107 ± 29 vs. 90 ± 28, *P* = 0.047) and aortic occlusion time (min) (111 (IQR 81–127) vs. 87 (IQR 72–116), *P* = 0.010) were significantly longer in the brain injury group than in the normal group. Patients in the brain injury group had greater decreased rSO_2_ (%) (27.0 ± 7.3 vs. 17.5 ± 6.1, *P* < 0.001) and cerebral blood flow (%) (44.9 (IQR 37.8–69.2) vs. 29.1 (IQR 12.0–48.2), *P* = 0.004) levels. Multivariate logistic regression analysis suggested that decreased rSO_2_ and cerebral blood flow levels, aortic occlusion time, and history of atrial fibrillation were independent risk factors for early postoperative brain injury (*P* < 0.05). ROC analysis reported that the best cutoff values for predicting early postoperative brain injury were 21.4% and 37.4% for decreased rSO_2_ and cerebral blood flow levels, respectively (*P* < 0.05).

**Conclusion:**

The decreased rSO_2_ and cerebral blood flow levels, aorta occlusion time, and history of atrial fibrillation were independent risk factors for early postoperative brain injury. TCCD and rSO_2_ could effectively monitor brain metabolism and cerebral blood flow and predict early postoperative brain injury.

## Introduction

Cardiopulmonary bypass (CPB) has been widely used in cardiovascular surgery since 1953, but CPB may also cause subsequent organ damage (e.g., brain damage) [1]. Injury to the central nervous system after cardiovascular surgery can reduce the quality of life of patients and lead to death [2], and the incidence of central nervous system complications after cardiovascular surgery could be as high as 5% [3]. Therefore, the use of perioperative brain monitoring equipment is conducive to the early detection of brain injury to reduce the incidence of brain injury and improve the prognosis of patients. The regional cerebral oxygen saturation (rSO_2_) reflects the oxygen supply and demand of brain tissues. Although rSO_2_ has been widely used to monitor brain oxygen metabolism during cardiovascular surgery [4–7], the intraoperative prediction of early postoperative brain injury has not been reported thus far. In addition, there is a lack of methods to effectively monitor the cerebral blood flow, which corresponds to cerebral perfusion during surgery. The transcranial color Doppler (TCCD) combines color Doppler, two-dimensional grayscale images, and spectral Doppler to display the blood vessels and determine the blood flow spectrum. Herein, this study aimed to use TCCD to monitor the changes in cerebral blood flow during cardiovascular surgery. Additionally, this study evaluated the risk factors of early brain injury after cardiovascular surgery and investigated the predictive value of TCCD and rSO_2_ for detecting early postoperative brain injury.

## Materials and methods

### Patients and study design

This study was approved by the Ethics Committee of The Affiliated Changzhou No. 2 People’s Hospital of Nanjing Medical University ([2022]KY216-01) and registered with the Chinese Clinical Trials Registry (ChiCTR2300072145). A total of 55 patients undergoing cardiovascular surgery with cardiopulmonary bypass at our hospital between October 2022 and August 2023 were selected as subjects for this study.

Inclusion criteria included the following: (1) patients undergoing cardiovascular surgery with cardiopulmonary bypass; (2) over 18 years old; (3) signed informed consent.

Exclusion criteria were as follows: (1) abnormal preoperative NSE concentration; (2) preoperative examination reported carotid artery stenosis; (3) reported stroke or transient ischemic attack within six months before surgery; (4) missing temporal window; (5) under 18 years old; (6) emergency operation; (7) patient rejection; and/or (8) unforeseen accidents during the perioperative period.

**Fig. 1:**
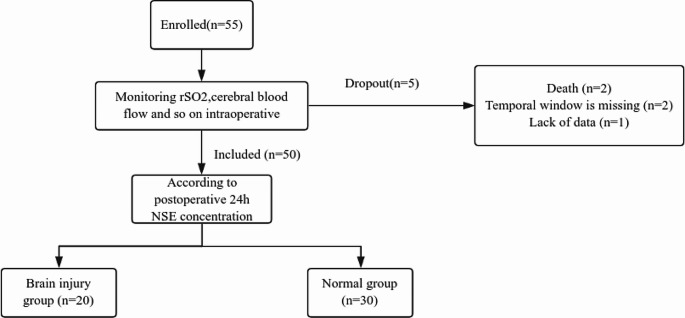
Flowchart of patient selection

The neuron-specific enolase (NSE) concentration reflects the degree of brain injury [8]. After excluding samples with serum Hb > (0.6 g/L) 24 h after surgery, the patients were divided according to the measured NSE concentration into the brain injury (≥ 16.3 ng/mL) and normal (0–16.3 ng/mL) groups. The flow chart of the study is displayed in Fig. 1.

### Anesthesia

Anesthesia monitoring, electrocardiogram, pulse oxygen saturation, and non-invasive arterial blood pressure were performed/measured after the patient entered the operating room. A radial artery puncture revealed the invasive arterial pressure. A three-lumen tube was placed into the right internal jugular vein, and the central venous pressure was measured. Intravenous anesthesia (midazolam at 0.1 mg/kg, propofol at 2 mg/kg, sufentanil at 1 µg/kg, and rocuronium at 1 mg/kg) was administered after 3 min of pure oxygen mask ventilation at 6 L/min with a tidal volume of 6–8 mL/kg and frequency of 16 times/min. The position of the tracheal tube was set based on the carbon dioxide waveform after expiratory breath. During the operation, norepinephrine, ephedrine, atropine, nitroglycerin, and dopamine were used to regulate blood pressure to the optimum. Transesophageal ultrasound was used to monitor the patient’s heart condition throughout the operation. A cardiopulmonary bypass was performed by the same person.

### TCCD measurement

A cardiac ultrasound instrument (Mindray, MT3, China) and a probe (P4-2s) with a probe frequency of 2.5 MHz were used to measure the bilateral middle cerebral artery flow. The probe was applied to the preauricular region of the temporal bone, and the optimal contralateral cranial window was scanned in B-scan mode to detect the midbrain. Color mode was then activated, and the colored image was superimposed on the B-scan image to display the middle cerebral artery. The spectrum was then generated with the angle between the direction of the ultrasound and the blood adjusted to less than 20°. The optimal spectrum was obtained, and the mean cerebral blood flow velocity (Vmin), diameter R of the middle cerebral artery, and blood flow velocity time integral (VTI) were measured accordingly. The cerebral blood flow per unit of time was calculated according to the formula: Q = π(R/2)^2×VTI

$$\text{Q}={\uppi }{(R/2)}^{2}\times \text{V}\text{T}\text{I}$$

The definitions of the relevant parameters are as follows:

Baseline cerebral blood flow value of each patient: The mean left and right cerebral blood flow were measured 5 min before anesthesia induction.

Minimum cerebral blood flow value of each patient: The left and right cerebral blood flow values were recorded every 10 min from the beginning to the end of the cardiopulmonary bypass. The average values of the left and right sides were calculated, and the lowest value was selected.

Decreased cerebral blood flow value: (Basic value of cerebral blood flow–minimum value of cerebral blood flow)/basic value of cerebral blood flow ×100%.

### rSO_2_ measurements

Intraoperative rSO_2_ was monitored by near-infrared spectroscopy (Covidien, 5100 C, USA). The level of rSO_2_ was continuously measured after the patient entered the operating room by attaching the electrodes to the left and right frontal parts of the patient.

The definitions of the relevant parameters are as follows:

Baseline rSO_2_ value of each patient: Average rSO_2_ readings on the left and right sides of the patient at the time of air inhalation measured 5 min before anesthesia induction.

Minimum rSO_2_ value of each patient: The average rSO_2_ value of the left and right sides was recorded every 10 min during cardiopulmonary bypass, and the lowest value was taken.

Decreased rSO_2_ value: (rSO_2_ base value–rSO_2_ lowest value)/rSO_2_ base value ×100%.

### Observational indices

The following indices were recorded and compared between the two groups: (1) general patient information such as age, sex, body mass index (BMI), history of hypertension, diabetes, cerebral infarction, and atrial fibrillation; (2) left ventricular ejection fraction (LVEF), preoperative platelet count, preoperative erythrocyte specific volume (HCT), operation time, cardiopulmonary bypass time, aortic occlusion time, postoperative hospital stay, NSE concentration, and other clinical indices; (3) decreased cerebral blood flow and rSO_2_ levels.

### Statistical analysis

IBM SPSS Statistics 23 (IBM Corporation, Armonk, NY, USA) was used for data processing, and the graphs were plotted using GraphPad Prism 8 (GraphPad Software, La Jolla, CA, USA). The Shapiro-Wilk test was used to test data normality, and the continuous variables were expressed as the mean and standard deviation (normal distribution) or the median and interquartile range (non-normal distribution). Differences were compared using the student’s t-test and Mann-Whitney U rank-sum tests. Categorical variables were expressed as numbers and percentages, and the differences were compared using a chi-square test. A multiple linear regression model was constructed to evaluate the relationship between perioperative data and NSE concentration. A logistic regression (step forward, enter when *P* < 0.05, exit when *P* > 0.1) model was constructed to determine the risk factors for early brain injury after cardiac surgery. The best predictive values for continuous variables were calculated based on recipient operating characteristic (ROC) curves, and both the Hosmer and Lemeshow statistics were used to assess the model fit. *P*-value < 0.05 was considered statistically significant.

## Results

A total of 55 patients were enrolled in the study, of which five were removed (two perioperative deaths, two missing temporal window, and one missing data), and 50 patients were included in the final analysis. A total of 20 patients had abnormal NSE (brain injury group), and a total of 30 patients had normal NSE (normal group).

### Comparison of baseline and perioperative data between the two groups

No statistically significant difference existed in basic features between the two groups (Table 1). For perioperative characteristics, CPB time (min) in the brain injury group was longer than that in the normal group (107 ± 29 vs. 90 ± 28, *P* = 0.047), and aortic occlusion time was significantly different between the two groups (*P* = 0.010), which was 111 (IQR 81–127) min in the brain injury and 87 (IQR 72–116) min in the normal group, respectively. In addition, the brain injury group reported lower rSO_2_ (%) (27.0 ± 7.3 vs. 17.5 ± 6.1, *P* < 0.001) and cerebral blood flow (%) (44.9 (IQR 37.8–69.2) vs. 29.1 (IQR 12.0–48.2), *P* = 0.004) values and larger concentrations of NSE (36.7 (IQR 30.2–49.3) vs. 10.6 (IQR 3.8–12.4), *P* < 0.001) than the control group (Table 2).

**Table 1:** Comparison of the basic characteristics between the two groups

Parameters	All patients (*N* = 50)	Brain injury group (*N* = 20)	Normal group (*N* = 30)	*P*-value^*^
Sex				1.000
Male, N	27 (54)	11 (22)	16 (32)	
Female, N	23 (46)	9 (18)	14 (28)	
Age (years)	57 ± 12	59 ± 7	56 ± 14	0.344
BMI (kg/m^2^)	24.3 ± 2.6	24.4 ± 2.5	24.1 ± 2.7	0.720
History of hypertension, N (%)	26 (50)	11 (22)	15 (30)	0.779
History of diabetes, N (%)	19 (38)	9 (18)	10 (20)	0.553
History of cerebral infarction, N (%)	11 (22)	7 (14)	4 (8)	0.09
History of atrial fibrillation, N (%)	21 (42)	11 (22)	10 (20)	0.154
LVEF (%)	54 ± 6	54 ± 5	55 ± 6	0.376
Preoperative platelet count	216 ± 79	201 ± 62	226 ± 89	0.291
Preoperative HCT	40.4 (34.2–43.0)	38.5 (34.7–42.8)	41.0 (33.0–43.0)	0.774

Data are expressed as mean ± standard deviation, median (interquartile range), or frequency (percentage). *P* < 0.05 was considered statistically significant. BMI: body mass index; LVEF: left ventricular ejection fraction; HCT: erythrocyte volume; *p-value indicates the comparison between the two groups

**Table 2:** Comparison of the perioperative characteristics between the two groups

Parameters	All patients (*N* = 50)	Brain injury group (*N* = 20)	Normal group (*N* = 30)	*P*-value^*^
CPB time (min)	97 ± 29	107 ± 29	90 ± 28	0.047
Aortic occlusion time (min)	60 (44–74)	111 (81–127)	87 (72–116)	0.010
Decreased rSO_2_ value (%)	21.3 ± 8.0	27.0 ± 7.3	17.5 ± 6.1	< 0.001
Decreased cerebral blood flow value (%)	38.0 (21.3–56.2)	44.9 (37.8–69.2)	29.1 (12.0–48.2)	0.004
Postoperative hospital stay (days)	14 (11–16)	14 (12–20)	13 (9–16)	0.064
NSE concentration (ng/mL)	13.4 (9.4–35.6)	36.7 (30.2–49.3)	10.6 (3.8–12.4)	< 0.001

Data are expressed as mean ± standard deviation, median (interquartile range), or frequency (percentage). *P* < 0.05 was considered statistically significant. *p-value indicates the comparison between two groups

### Correlation of cerebral blood flow, rSO_2_, and NSE concentration

The variance inflation factor (VIF) of all continuous variables was less than five, indicating that there was no interaction between the continuous variables. Baseline data and perioperative characteristics were included in the variables to construct a linear regression model for NSE concentration. Results indicated that the decreased rSO_2_ levels strongly correlated with the concentration of NSE (β = 0.583, *P* < 0.001). In addition, NSE concentrations were higher in patients with a history of cerebral infarction (β = 0.279, *P* = 0.020) (Table 3).

To determine the variables related to abnormal NSE concentration, the decreased rSO_2_ effect was excluded from the variables, and the remaining variables were included in another multiple linear regression analysis. Results revealed that the regression equation of this model was significant (F = 5.746, *P* < 0.001) (Table 4). NSE concentration was lower in patients without a history of cerebral infarction than in patients with a history of cerebral infarction before surgery (β = 0.289, *P* = 0.027). Lower cerebral blood flow led to higher NSE concentrations (β = 0.354, *P* = 0.005). Similarly, aortic occlusion time (β = 0.332, *P* = 0.011) effectively predicted NSE concentration. Taken together, these variables accounted for approximately 40.4% of the differences in NSE concentrations between the two groups.

**Table 3:** Analysis of variables potentially associated with NSE concentration (including rSO_2_)

Parameters	β	*P*-value	F	Adjusted *R*^2^
History of cerebral infarction	0.279	0.020	8.794^***^	0.488
Decreased rSO_2_ level	0.583	0.000		

***, statistically significant; *P* < 0.05, statistically significant

**Table 4:** Analysis of variables potentially associated with NSE concentration (excluding rSO_2_)

Parameters	β	*P*-value	F	Adjusted *R*^2^
History of cerebral infarction	0.289	0.027	5.746^**^	0.404
Decreased cerebral blood flow value	0.354	0.005		
Aortic occlusion time	0.332	0.011		

***, statistically significant; *P* < 0.05, statistically significant

### Analysis of multivariate logistic regression results

In the regression model that included the decreased rSO_2_ level (Table 5), decreased rSO_2_ decline was an independent risk factor for postoperative brain injury, with an odds ratio value (OR) of 1.246. The regression model that did not include the decreased rSO_2_ level (Table 6), the decreased cerebral blood flow (OR = 1.047, *P* = 0.013), the duration of aortic occlusion (OR = 1.044, *P* = 0.010), and the history of atrial fibrillation (*R* = 5.257, *P* = 0.044) were independent risk factors for postoperative brain injury. The Hosmer-Lemeshow test revealed that the model was well-fitting (*P* > 0.05).

**Table 5:** Predictors of brain injury in multiple logistic regression (including rSO_2_)

Parameters	OR value	95% CI	*P*-value	
Decreased rSO_2_ level	1.246	1.092–1.421	< 0.001	

OR value: Odds ratio; CI: Confidence interval

**Table 6:** Predictors of brain injury in multiple logistic regression (excluding rSO_2_)

Parameters	OR value	95% CI	*P*-value	
Decreased cerebral blood flow level	1.047	1.011–1.084	0.013	
Aortic occlusion time	1.044	1.009–1.080	0.010	
History of atrial fibrillation	5.257	1.049–26.356	0.044	

OR value: Odds ratio; CI: Confidence interval

### Analysis of the best predictive values in the ROC curve

**Fig. 2:**
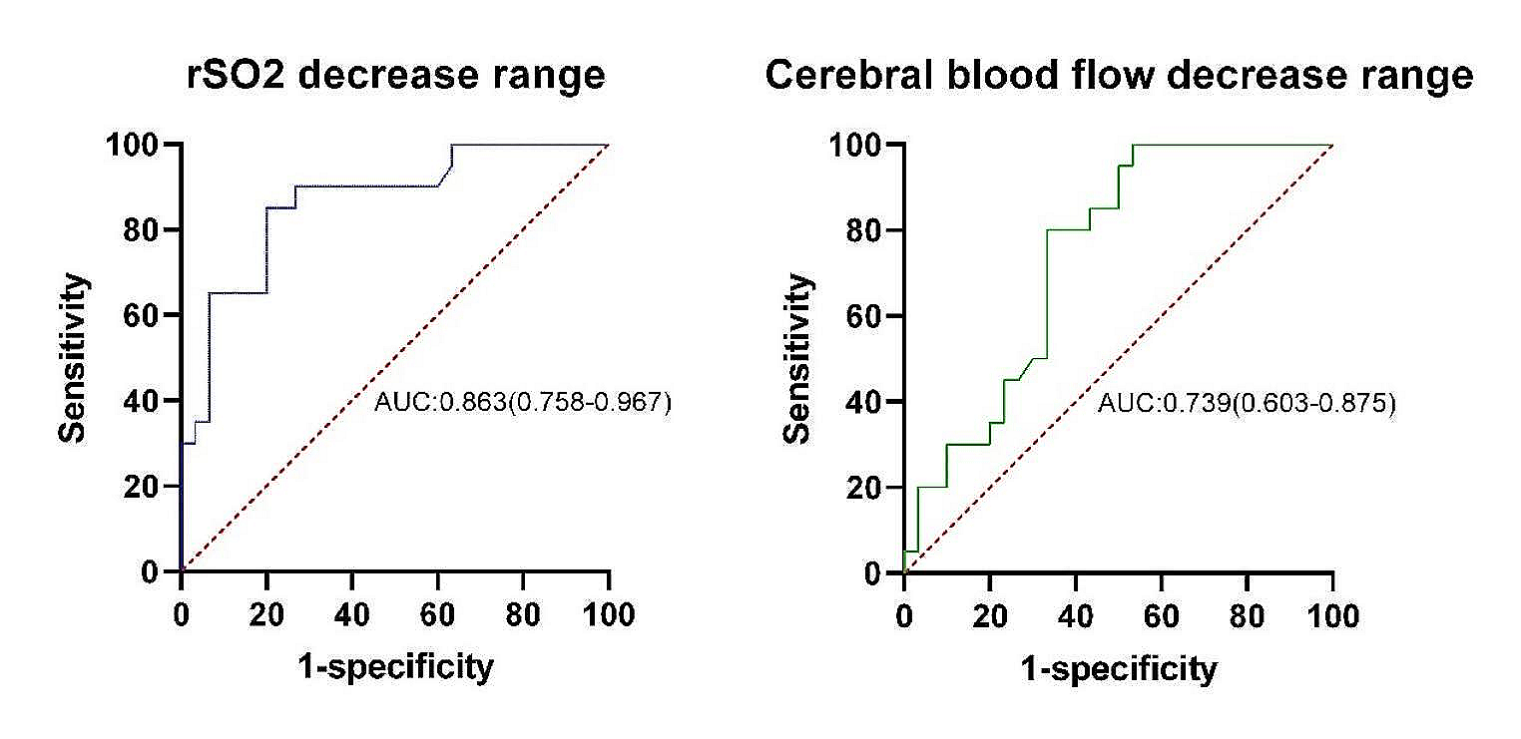
The area under the ROC curve for the ability of decreased rSO_2_ and cerebral blood flow levels to predict early brain injury

The area under the ROC curve, corresponding to the correlation between decreased rSO_2_ and brain injury, was 0.863 (95% CI: 0.758–0.967, *P* < 0.001). The area under the ROC curve, corresponding to the correlation between decreased cerebral blood flow and brain injury, was 0.739 (95% CI: 0.603–0.875, *P* = 0.004) (Fig. 2). The best intercept values for predicting brain injury were 21.4% (sensitivity: 80%; specificity: 85%) and 37.4% (sensitivity: 67%; specificity: 80%) for decreased rSO_2_ and cerebral blood flow levels (Table 7).

### Comparison of postoperative hospital stay between the two groups

**Fig. 3:**
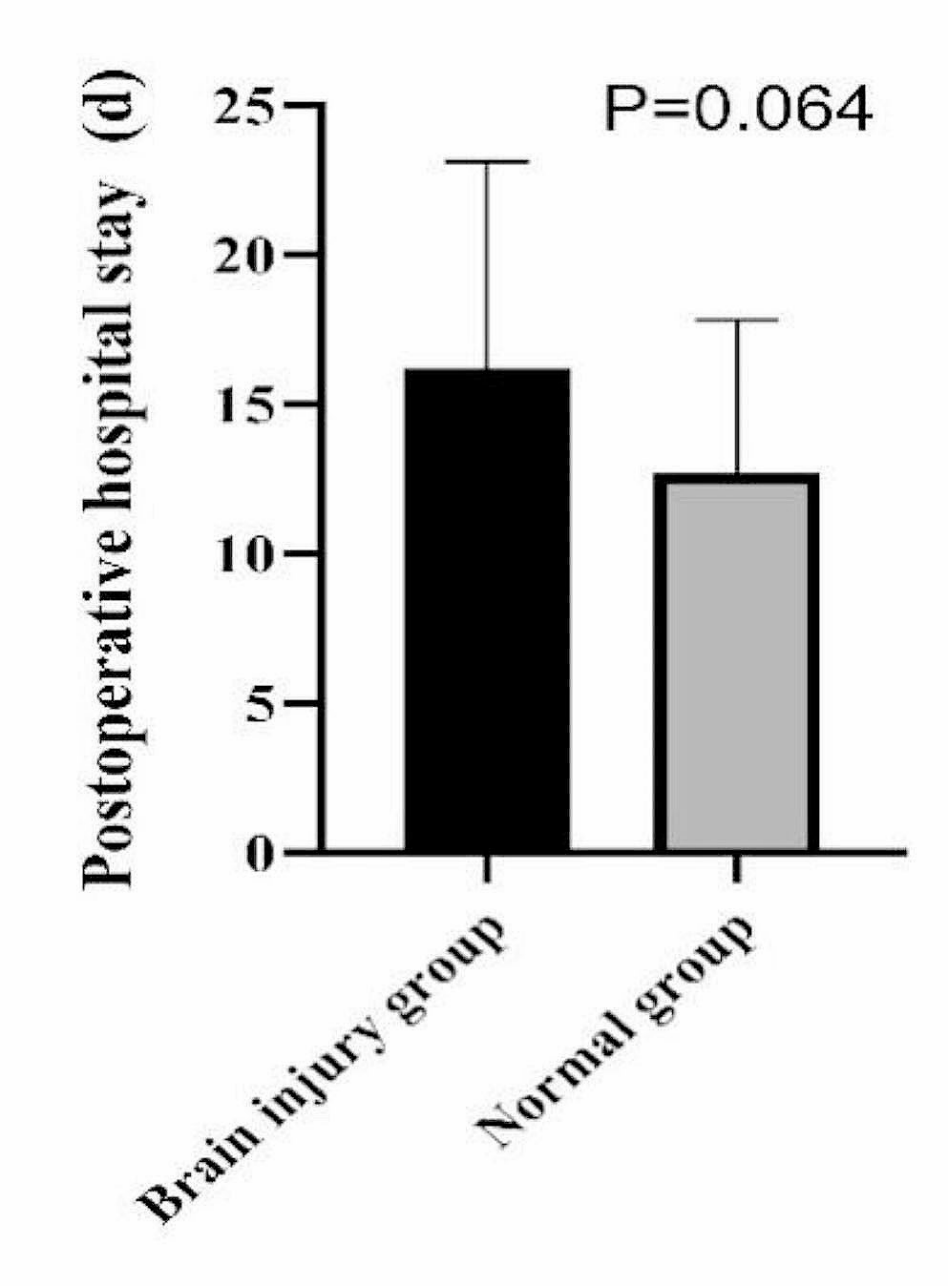
Comparison of postoperative hospital stay between the brain injury and normal groups

Patients in the brain injury group reported a longer hospital stay than patients in the normal group, but the difference was not statistically significant (*P* = 0.064) (Fig. 3).

**Table 7:** The ability of decreased rSO_2_ and cerebral blood flow levels to predict early brain injury

Parameters	AUC	*P*-value	95% CI	sensitivity	specificity	Cutoff value
Decreased rSO_2_ level	0.863	< 0.001	0.758–0.967	80%	85%	21.4%
Decreased cerebral blood flow level	0.739	0.004	0.603–0.875	67%	80%	37.4%
Aortic occlusion time	0.717	0.010	0.570–0.863	40%	96.7%	86

AUC: Area under the curve

**Fig. 4:**
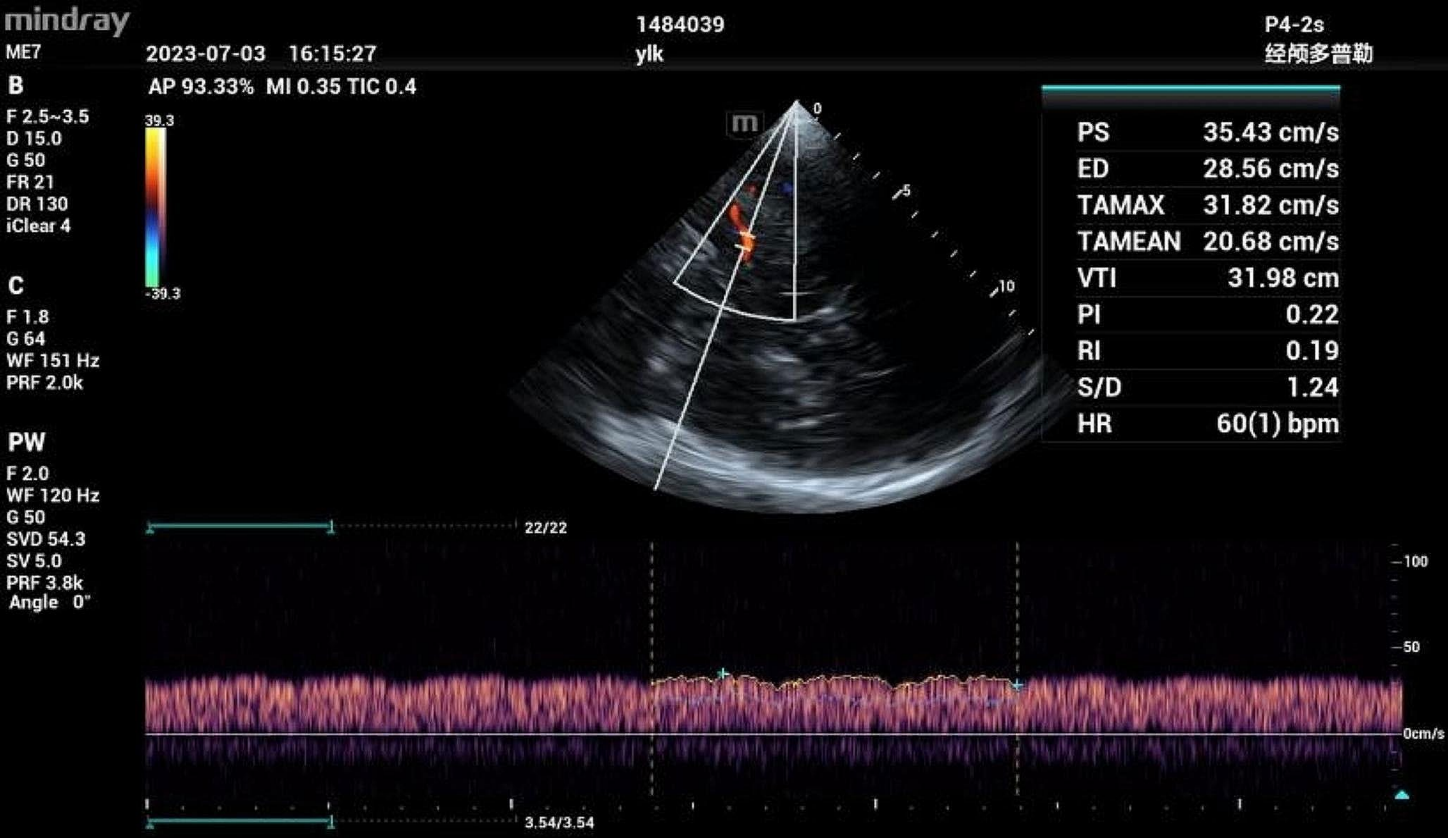
Cerebral blood flow spectrum during cardiopulmonary bypass

## Discussion

The non-pulsating blood flow caused by the cardiopulmonary bypass device damages multiple organs in the body, especially the brain. Non-physiological blood flow alters blood supply to the brain tissues, and damage to the transmission of arterial pulsation to the “pulse brain” tissue will alter the brain structure and function [9, 10]. Brain injury after cardiovascular surgery will increase the degree of pain in patients and reduce their quality of life [2]. Therefore, it is crucial to implement intraoperative brain monitoring and timely intervention to reduce the incidence of postoperative brain injury. In this study, TCCD was used to monitor the changes in cerebral blood flow during cardiac surgery, and rSO_2_ was used to monitor the changes in cerebral oxygen metabolism. Taken together, TCCD and rSO_2_ could effectively predict early postoperative brain injury, and this study also identified the risk factors of early brain injury after cardiovascular surgery.

NSE is considered a specific neuro-biochemical marker of neuronal injury [11]. The level of serum NSE was significantly increased after brain injury and was significantly correlated with the severity of brain injury after ischemia [12]. In this study, the patients were divided into the early postoperative brain injury and normal groups according to the measured NSE concentration 24 h post-surgery. We observed significant differences in CPB time, decreased rSO_2_ level, decreased cerebral blood flow level, aortic occlusion time, and NSE concentration between the two groups. Further analysis indicated that decreased rSO_2_ level was strongly correlated with NSE concentration. In the model without decreased rSO_2_, decreased cerebral blood flow level, aortic occlusion time, and cerebral infarction history were correlated with NSE. Therefore, decreased rSO_2_ was considered the most important factor affecting NSE concentration. Patients with a history of cerebral infarction were more likely to be affected by ischemia, and the degree and time of low cerebral blood flow that could be tolerated were also reduced. Hence, patients with a history of cerebral infarction in this trial were more likely to develop postoperative NSE abnormalities.

The parameter rSO_2_ has good stability and is not affected by arterial pulse. Additionally, relatively stable rSO_2_ values could be observed in patients with hemodynamic instability or even cardiac arrest [13]. In this study, decreased rSO_2_ level was identified as an independent risk factor for postoperative brain injury with high diagnostic value. If decreased rSO_2_ level was more than 21.4%, postoperative brain injury was more likely to occur. Similarly, Klinkova et al. observed that 19% of 200 patients with a 20% or greater decrease in rSO_2_ during CPB had a higher risk of neurological complications [14]. It was also reported that maintaining intraoperative rSO_2_ levels above 80% of the baseline value during cardiac surgery could reduce the incidence of cognitive impairment seven days post-surgery [15]. In a coronary artery bypass grafting study, patients with rSO_2_ levels maintained above 75% of baseline value spent significantly less time in the intensive care unit and hospital stay after surgery than patients in the no-intervention group [16]. These studies demonstrated that monitoring rSO_2_ is important to improve cardiac surgery. However, rSO_2_ levels may be affected inevitably. rSO_2_ monitoring is affected by the position of its electrodes. Usually, the electrodes are selected to be placed on the forehead. Although global cerebral oxygen sufficiency can be evaluated, it may not be possible to detect changes in areas far from the monitored site. Another factor is its signal may be disturbed by external information, such as skull thickness, skin pigment, etc., and we may eliminate some of the effects by maintaining the same position during the operation. Moreover, rSO_2_ monitoring is also susceptible to hemoglobin concentration, especially during cardiac surgery, when blood is diluted to a certain extent, such that decreased hemoglobin levels can also lead to decreased rSO_2_ levels [17]. Therefore, it is inadequate to evaluate cerebral blood flow with only rSO_2_ levels during surgery. Likewise, this study further evaluated TCCD to measure cerebral hemodynamics, obtain cerebral blood flow, observe vascular misalignment, and determine the presence of plaque [18]. Our findings revealed that, in the absence of decreased rSO_2_ levels, decreased cerebral blood flow (measured by TCCD) was identified as an independent risk factor for early postoperative brain injury, and the decrease of more than 37.4% could predict early postoperative brain injury. Six patients in the brain injury group had no significant decrease in rSO_2_, while the decrease in cerebral blood flow measured by TCCD was much greater than 37.4%. Hence, TCCD could be used to supplement rSO_2_ monitoring for more accurate and comprehensive results. According to Catena et al. [19], during aortic arch surgery, TCCD could monitor cerebral blood flow, optimize the rate of anterograde and retrograde cerebral perfusion, and avoid hyper-perfusion or hypo-perfusion during cardiac arrest. Additionally, during cardiopulmonary bypass, the cerebral blood flow spectrum was altered with a jagged waveform, which was consistent with our observation (Fig. 4). This change is related to the use of non-pulsating pumps during cardiopulmonary bypass. In addition, TCCD can also detect the occurrence of embolic events [20]. However, its limitations can lead to difficulties. TCCD operation is dependent on the operator’s proficiency. Therefore, it is not easy for any one person to obtain it, provided that it is systematically trained.

In cardiovascular surgery, it is often necessary to block the aorta to facilitate the operation. However, the results of this study suggested that the duration of aortic occlusion over 86 min may indicate early postoperative brain injury. Long-term aortic occlusion may not only affect cerebral perfusion but also damage the vascular endothelium and induce coagulation disorders. In addition, a history of atrial fibrillation was a risk factor for early postoperative brain injury. Atrial fibrillation causes cerebral blood flow and fluctuations in blood perfusion to the brain. Studies have found that atrial fibrillation can damage the blood-brain barrier, causing patients to be more susceptible to cognitive impairments later in life [21]. In this study, the duration of hospital stay in the brain injury group was longer than that in the normal group, but there was no statistical difference. However, in some studies, longer hospital stays after surgery were associated with lower rSO_2_ levels [4, 22], which could be due to other clinical factors.

There were some limitations in this study, particularly the inclusion of more than one type of cardiovascular surgery, which might have affected the results. Likewise, further research is required to investigate specific interventions to reduce early postoperative brain injury.

In conclusion, the combined multi-modal monitoring of rSO_2_ and TCCD holds great significance for brain protection during cardiovascular surgery and can be used to predict early postoperative brain injury.

## Data Availability

No datasets were generated or analysed during the current study.
